# Training Does Not Uniformly Increase Canine Self-Control

**DOI:** 10.3390/ani15030320

**Published:** 2025-01-23

**Authors:** Zachary A. Silver, Rebecca A. Singer, Riley Welch, Isabella Perry, Caitlyn D. Jones, Ahna Cates, Camden Foster, Sydney Sullivan, Isla Hall, Ellen E. Furlong

**Affiliations:** 1 Department of Psychology, Occidental College, Los Angeles, CA 90041, USA; cfoster@oxy.edu (C.F.); ssullivan2@oxy.edu (S.S.); ihall@oxy.edu (I.H.); 2 Department of Psychology, Berea College, Berea, KY 40403, USA; singerr@berea.edu (R.A.S.);; 3 Department of Psychology, Georgetown College, Georgetown, KY 40324, USA; rileyw@georgetowncollege.edu (R.W.); iperry@georgetowncollege.edu (I.P.); 4 Department of Psychology, Transylvania University, Lexington, KY 40508, USA; ahna.cates@gmail.com

**Keywords:** self control, impulse control, canine cognition, training differences

## Abstract

Dog trainers make widespread claims that training dogs to improve self-control is essential to solving behavior problems in domesticated dogs. We investigated whether one aspect of self-control, motor inhibition, covaries in dogs with varying levels and contexts of formal training. We tested pet dogs with little formal training and dogs with high levels of training who compete in scent work, barn hunt, and agility. All dogs were tested for motor inhibition on the cylinder task, a validated self-control task. First, in familiarization trials, dogs watched a piece of food placed inside an opaque cylinder and were subsequently allowed to retrieve it. Then, in test trials, dogs watched a piece of food placed inside a transparent cylinder. This transparent variation of the task requires dogs to inhibit the response to directly approach the now-visible food and instead detour to an open side. In contrast to the belief that highly trained dogs would uniformly demonstrate better self-control, trained dogs did not perform better than untrained dogs. In fact, dogs who competed in barn hunt performed significantly worse compared to untrained pet dogs. These findings suggest that self-control may involve a complex set of mental processes that do not uniformly generalize to multiple contexts.

## Simple Summary

Dog trainers make widespread claims that training dogs to improve self-control will solve a variety of behavior problems. However, these claims have not been directly tested. In the current experiment, we investigated whether dogs with formal training would perform better on one type of self-control task than pet dogs. We compared dogs trained in scent work, barn hunt, and agility to pet dogs on a task in which dogs had to detour to the side of a clear tube to obtain a food reward. Dogs first learned to obtain food from an opaque cylinder. We tested their ability to exert self-control by then placing the food treat inside a transparent cylinder. Dogs with better self-control should detour to the side rather than approaching the food directly. Contrary to the claims of dog trainers, highly trained dogs did not perform any better than untrained pet dogs. These findings suggest that self-control may involve a complex set of mental processes that do not generalize across multiple settings. There is a need for future research into what, if any, training can improve self-control in domesticated dogs.

## 1. Introduction

Behavioral problems account for 40% of pet dog relinquishments, with bites, aggression, escaping, and destructiveness among the most commonly reported problem behaviors [1,2]. While trainers have developed many strategies to help approach these problematic behaviors, recently they have focused on exploring the role of inhibitory control, on the assumption that inhibitory control can regulate and reduce these common causes of relinquishment, e.g., [3,4,5,6,7,8]. Trainers and organizations suggest that self-control can be improved by working on specific behaviors such as wait, leave it, and off, or perfecting a long sit and stay [4,7,8].

However, while these trainers imply that inhibitory control acts as a domain-general skill, that is, that training it in one context spreads to other contexts, the current literature challenges this view, suggesting that canine inhibitory control is instead extremely context specific. Bray and colleagues [9] examined the correlation between canine performance in a three-task inhibitory control battery. Dogs’ performances failed to correlate across tasks. That is, dogs who performed well on one inhibitory control task did not necessarily perform well on any of the others. This suggests that inhibitory control is not a singular domain-general trait determining dogs’ performance across varying tasks, as many trainers seem to suggest.

Rather than possessing a baseline level of executive functioning that predicts performance on all inhibitory control tasks, dogs may adapt their inhibitory control abilities to specific task demands. Brucks et al. [10] suggest that self-control may involve several different facets, such as persistence, compulsivity, and decision time. These different variables may account for the lack of correlation in tasks, including owner reports of self-control [11]. In a recent review, Foraita and colleagues [12] identified experiential and environmental influences that impact executive function in dogs and identified three factors that influenced inhibitory control: early life experiences, housing, and training. The role of training on executive function in dogs has been studied in a variety of contexts with mixed results [13]. 

Research suggests that specific types of training enhance certain skills without universally improving cognitive traits. For example, clicker-trained dogs solved puzzles quicker and with fewer errors than their untrained counterparts [14]. Dogs trained for diverse tasks, such as agility, also showed an advantage, solving puzzle boxes more frequently than untrained dogs [15]. Additionally, behavioral differences are seen in police dogs, as some evidence suggests they exhibit reduced self-control compared to untrained dogs [16]. While we do not know if these differences exist because of training broadly or because of specific training practices (that is, positive reinforcement training vs. aversive methods), it does appear that training at least correlates with some cognitive differences.

Gobbo and Zupan Šemrov [16] assessed self-control in police dogs using a delayed gratification test. Dogs scored higher on self-control if they waited for high-quality food instead of immediately eating the low-quality food. Cognitive inhibition was measured using a reversal learning test, which required the dog to inhibit a previously learned response and shift their response to a new reward pattern. Police dogs exhibited poorer self-control compared to other dogs on the delay of gratification test but not the cognitive inhibition task. The mixed results in dogs’ performance on self-control and cognitive inhibition tasks suggest that these tasks measure distinct mental processes and are therefore not interchangeable.

Mellor et al. [17] also found that training affected inhibitory control in dogs. Dogs trained in scent work, agility, and obedience were tested on two different self-control tasks. Mellor and colleagues [17] predicted that scent work dogs, who are trained to work in highly distracting and high-arousal environments requiring focused attention on specific stimuli while ignoring other stimuli, would outperform agility and obedience dogs. While individual task performance did not differ across disciplines, a principal components analysis (PCA) showed that inhibitory control was highest for scent-trained dogs. Similarly, Barrera et al. [18] compared highly trained water rescue dogs to pet dogs on the A-not-B task. Water rescue dogs made significantly fewer errors than untrained pet dogs.

We aim to provide a preliminary exploration of the role of dog training on self-control by investigating whether specific types of training yield different impacts on canine self-control. We tested dogs from four distinct training backgrounds (dogs trained to compete in agility competitions, dogs trained to compete in barn hunt competitions, dogs trained to compete in scent work competitions, and pet dogs with no formal training) on a widely used self-control task, the cylinder task. Because the context in which dogs were trained varies across the four groups, but the context in which they must demonstrate their self-control is held constant (the cylinder task), we can attribute any differences in performance across the training groups to their training background.

## 2. Method

### 2.1. Participants

We tested 162 dogs (79 males, *M*_age_ = 4.91, *SD*_age_ = 3.29) from the central Kentucky region. We recruited pet dogs with no particular training and dogs who were advanced in training enough to compete in one of three dog sports (agility, barn hunt, scent work). This included 78 dogs who competed in agility (38 males, *M*_age_ = 5.73, *SD*_age_ = 3.57), 21 dogs who competed in barn hunt (7 males, *M*_age_ = 3.48, *SD*_age_ = 2.39), 14 dogs who competed in scent work (10 males, *M*_age_ = 4.36, *SD*_age_ = 2.92), and 49 pet dogs with no particular training history (24 males, *M*_age_ = 4.38, *SD*_age_ = 2.95). Owners of 11 of the pet dogs reported that they competed in one or more sports. These dogs were excluded from further analyses.

Dogs represented most AKC breed groups, including 56 herding breeds, 29 sporting breeds, 29 terriers, 8 toys, and 5 hounds. We did not have many working or nonsporting dogs so we combined them to form a miscellaneous category that included 5 dogs. The remaining 30 dogs were mixed breeds (see Table 1 for breed breakdown by training group). We had data on the desexing status of 141 of the dogs. Most were desexed (*n* = 104).

### 2.2. Materials and Apparatus

We tested sports dogs either while competing at the Kentuckiana Cluster of Dog Shows (the agility dogs) or while taking a class at Bluegrass Dog Training Academy (the barn hunt and scent work dogs). Dogs were removed to a quiet room away from the noise of the competition or training. After testing they returned to their class or competition. Owners worked in testing their dogs between runs: some dogs had already completed their runs, others had not begun, and yet others were tested between runs. We do not have data on the time between their last run and testing. Pet dogs were tested in a dedicated on-campus laboratory at Transylvania University.

**Figure 1:**
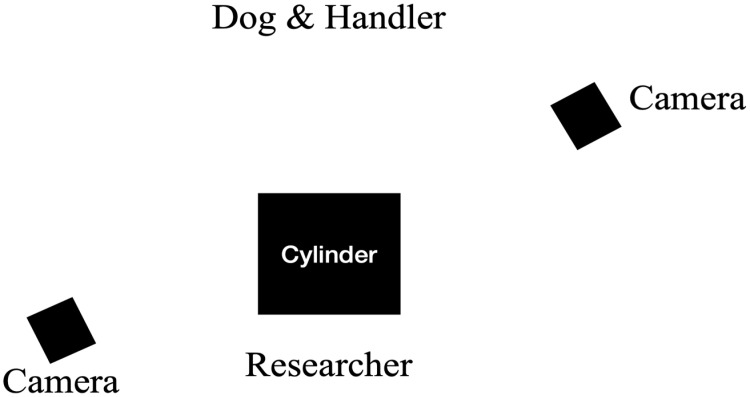
The setup of the testing layout. The dog watched as the researcher showed them food and placed it in the cylinder. The researcher then turned around and took several steps away from the cylinder before saying “Okay”. Once the researcher said “Okay”, the handler released the dog to approach the cylinder. Once the dog retrieved the food they returned to the start position for the next trial.

For all groups, owners served as handlers throughout the study. Dog-handler pairs stood facing the apparatus and one researcher. Two cameras on tripods were positioned to record the dog’s behavior: one recording the front of the apparatus, and one recording the side of the apparatus (see Figure 1 for the setup of the testing).

**Figure 2:**
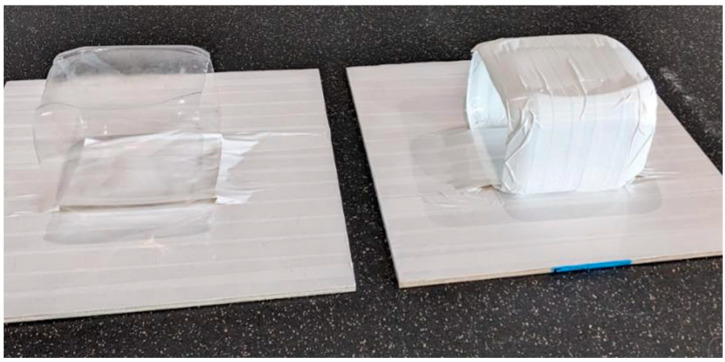
Photograph of the two testing apparatuses. The testing apparatus (**left**) and the training apparatus (**right**). Photo credit: R.A.S.

The apparatuses consisted of a plastic cylinder mounted on a piece of plywood (61 cm × 61 cm) for support. The training apparatus was opaque (covered in white duct tape; 23 cm from left to right and 20 cm from front to back) and the testing apparatus was identical except it remained transparent. Both were secured to the plywood base with white duct tape. Each cylinder was open on both sides (Figure 2).

### 2.3. Procedure

Dogs were introduced to the task with four familiarization trials using the opaque apparatus to acquaint them with the task. The handler and dog stood facing the opaque cylinder. The researcher stood behind the cylinder, showed the dog a treat, and then placed the treat inside the opaque cylinder. The experimenter then turned and walked a few steps away from the cylinder and gave the release cue “Okay!” to the handler. The handler then released the dog to retrieve the food. Some dogs hesitated to approach the cylinder on the first familiarization trial. In these cases, we encouraged their handler to approach the cylinder and stand near it, at which point dogs all retrieved the treat inside. After this they continued to the rest of the familiarization trials. All dogs successfully retrieved the food in all four familiarization trials and, therefore, all advanced to testing.

The familiarization trials were followed by six testing trials. The testing trials were identical to the training trials except the transparent cylinder replaced the opaque cylinder.

Dogs correctly solved this task if they immediately detoured to retrieve the food from the open side of the cylinder. If dogs first touched the top or front of the cylinder with their face or paw, as if attempting to retrieve the food without detouring, they failed the trial. We counted the number of trials dogs succeeded (out of 6) on the test trials and also explored dogs’ performance on the first trial alone.

## 3. Analysis and Results

### 3.1. Statistical Analysis

**Figure 3:**
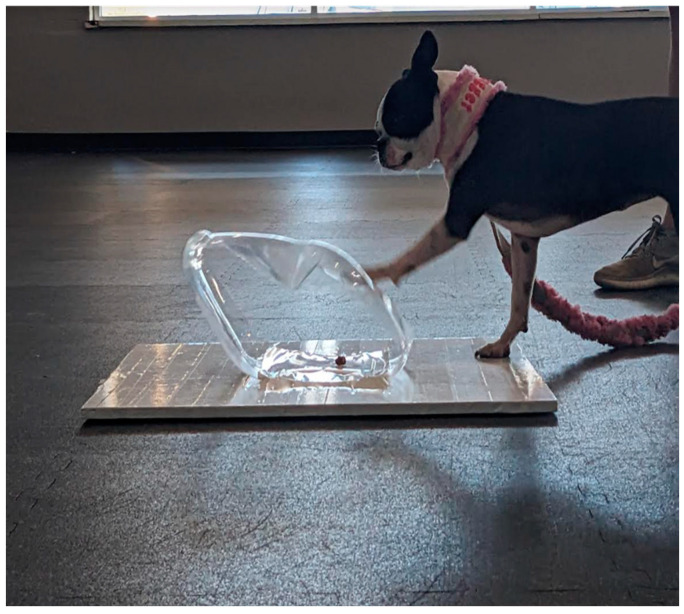
Photograph of barn hunt dog responding incorrectly to test trial by pawing at front of cylinder. Photo credit: R.A.S.

We measured dogs’ performance on the cylinder task by counting the number of times they successfully detoured around the cylinder to retrieve the food item without first touching the front or top of the cylinder with their nose or paw (see Figure 3 for an example of an incorrect response). Next, we explored the effect of learning by examining group differences in performance on the first trial of the cylinder task. Here, we scored the data a “1” if the dog correctly detoured on trial one and a “0” if the dog made an error. We next explored demographic effects on performance by examining sex differences, effects of desexing, and effects of breed on performance.

Levene’s test for the homogeneity of variances indicated that the assumption was violated for the number of correct detours, *F*(3, 145) = 4.09, *p* = 0.008; first-trial performance, *F*(3, 145) = 6.68, *p* < 0.001; and sex, *F*(1, 147) = 9.04, *p* = 0.003. We therefore used Welch’s test with Games–Howell post hoc tests where appropriate to explore these group differences. Our data did not violate the assumption of homogeneity of variance for desexing status or breed group and we therefore conducted ANOVAs for these analyses.

### 3.2. Results

We found a significant effect of the training group on cylinder task performance—*F*(3, 41.74) = 3.01, *p* = 0.041, *η*^2^ = 0.084—such that barn hunt dogs (*M* = 3.91, *SD* = 2.19) performed worse than pet dogs with no special training (*M* = 5.44, *SD* = 1.36, *p* = 0.003, *d* = 0.844). No other groups differed from each other (Table 2).

To explore whether this difference occurred due to learning, we then compared performance on the first trial across dog training groups but found no significant effect of training group on first-trial performance: *F*(3, 41.89) = 2.69, *p* = 0.059.

We did find a significant effect of sex on overall performance—*F*(1, 132.06) = 6.75, *p* = 0.010, *η*^2^ = 0.044—such that females (*M* = 5.30, *SD* = 1.135) outperformed males (*M* = 4.62, *SD* = 1.83). The effect of sex on performance, however, did not persist when looking at first-trial performance alone—*F*(1, 147) = 3.209, *p* = 0.075 (males: *M* = 0.64, *SD* = 0.48; females: *M* = 0.77, *SD* = 0.42).

We found no effect of desexing: desexed dogs (*M* = 4.95, *SD* = 1.70) did not perform differently than intact dogs overall—*M* = 4.70, *SD* = 1.70, *F*(1, 128) = 0.54, *p* = 0.465—nor did they perform differently at the first trial: *F*(1, 128) = 0.845, *p* = 0.36 (desexed: *M* = 0.72, *SD* = 0.45; in-tact: *M* = 0.64, *SD* = 0.49).

Similarly, we found no effect of breed on overall performance—*F*(6, 142) = 2.66, *p* = 0.39—or on first-trial performance, *F*(6, 142) = 1.03, *p* = 0.41.

## 4. Discussion

Our data suggest that formal training did not universally increase dogs’ performance on a classic measure of canine self-control, the cylinder task. In fact, dogs trained to compete in barn hunt competitions performed significantly worse on the cylinder task than untrained pet dogs. These data have several important implications for dog training, the empirical study of self-control, and canine wellbeing.

Dog trainers and dog training organizations promote training in self-control to improve problematic behaviors related to impulse control. In this initial study, we explored this by studying dogs who already had a significant amount of training and comparing them to dogs who did not. Though most dog trainers suggest improving self-control involves targeting cues such as a long stay in the face of distractions, a leave it, or a wait, we chose to study dogs who compete in a sport. We chose this population as a place to start addressing this question because all of the dogs who compete in these sports needed to have a good functional stay in order to compete. In fact, all of the dogs needed to be able to stay on cue until their handlers released them to engage in their sport: an activity that they find both highly arousing and highly rewarding. Therefore, we believe this population offered a good start to exploring the effects of training on self-control. We failed to find support for the claim that highly trained dogs performed better on our self-control task than pet dogs without such training.

Studies have documented how specific types of training enhance certain skills without universally improving cognitive traits. Previous research on training differences in canine cognition suggests that training appears to augment some facets of canine cognition adjacent to self-control [19]. To illustrate, clicker-trained dogs solved a puzzle more quickly and with fewer errors than their untrained counterparts [14]. In a puzzle box task, trained dogs from various agility and non-agility backgrounds solved the paradigm more frequently than untrained dogs [15]. Dogs trained for hunting purposes, or “gun dogs”, surpassed untrained dogs in following human pointing cues [20].

The enhanced success of trained dogs across various cognitive tasks may stem from their high attentiveness to humans—though our data cannot address this, we think this idea warrants further study based on some other research suggesting that training may encourage dogs to attend more to handlers. For example, compared to untrained dogs, Schutzhund-trained dogs looked at their owners more frequently when walking on a leash [21]. Within trained groups, agility dogs may be particularly attentive to humans. When presented with an unsolvable task, agility dogs spent longer looking for their owners than both untrained and search and rescue dogs [22]. Highly trained agility dogs also outperformed untrained pet dogs on a social evaluation task requiring dogs to seek out a helpful human over an unhelpful human [19]. One possible interpretation of this is that the agility dogs paid closer attention to the actions of the helpful and unhelpful human. While these data demonstrate that formal dog training may augment some elements of canine cognition, they do not directly support the belief commonly held by dog trainers that formal training should boost self-control [23]. In fact, research on generalization in both humans [24,25] and dogs [26,27,28] suggests that learning a complex skill like self-control in one domain may not generalize well to new ones.

In the present study, we did not find that highly trained dogs demonstrated better inhibitory control than untrained, pet dogs. In fact, contrary to research by Mellor et al. [17], we found that dogs trained on scent work performed no differently than pet dogs. Further, dogs trained in barn hunt (another scent work activity) did perform differently than pet dogs, but in the opposite direction to that predicted. Barn hunt dogs performed significantly worse at the cylinder task than untrained pet dogs. The way in which barn hunt dogs “failed” the cylinder task may explain their poor performance: instead of detouring when food was placed in a transparent cylinder, many of these dogs approached and pawed at the front of the cylinder. In barn hunt, when a dog finds a rat they often are trained to paw at the tube (Figure 3). Thus, it is possible that their barn hunt training interfered with their ability to successfully complete the cylinder task rather than augmenting it as we hypothesized.

Given this difference between barn hunt dogs and untrained dogs, we suspect that the barn hunt dogs’ response was context specific. That is, they responded to finding the food in the same way that they respond when locating the hidden rat in a barn hunt context (see Figure 3), by initiating physical contact with their paw. Thus, perhaps the reason barn hunt dogs performed significantly worse than pet dogs has nothing to do with self-control; rather, the specific methodology for testing self-control interfered with our assessment. The premise of context specificity in the assessment of self-control may also explain previous data suggesting that many established measures of canine self-control do not correlate with one another as, e.g., in [8]. Dogs’ performance on specific self-control tasks may reflect not only their actual self-control abilities but also their prior life experiences, training history, and the methodological constraints of the given task. Under this view, future research on canine self-control should seek to identify factors, such as the specific context of training history, that could predict dogs’ performance on the tasks used to assess self-control.

Given that most dog training is related to improving dogs’ performance in specific scenarios (e.g., decreasing the time to complete an agility course), the finding that training does not uniformly increase self-control suggests that to improve self-control, we should consider training self-control in the specific contexts for which we desire dogs to demonstrate effective self-control. In other words, we should not necessarily expect agility, scent work, or barn hunt training to result in universal improvements in self-control. Instead, we should approach training for self-control in a context-specific manner. This would involve training self-control in scenarios more specific to dogs’ natural environments (e.g., structured exposure to situations that often lead to problematic behaviors). That is, rather than expecting a dog with “wait” training to uniformly be able to apply this to any situation, we may need to train them to, say, wait before eating their dinner, wait before crossing a busy street, and wait before greeting someone at the door. This may more accurately reflect what trainers are actually doing when they report they are training “self-control”, but it does not reflect how they talk about this work.

Universally increasing self-control is likely not the intended outcome of the types of training we explored in the present study. However, success in agility, scent work, or barn hunt competitions requires some degree of context-specific self-control. Thus, our findings provide evidence that training protocols should be precisely tailored to the specific context in which dogs are likely to utilize the skills gained during training. That is, our data provide evidence against the belief that dogs can transfer self-control from the context in which it is trained into day-to-day situations.

Similarly, interpreting self-control as a highly context-specific phenomenon suggests the need to reconsider how we empirically test canine self-control. For example, our dogs typically performed exceptionally well on the cylinder task: most dogs scored 5 out of 6 or better on the task. Therefore, the task may not accurately reflect the full variability in self-control in dogs. Further, our existing methods largely measure self-control in contexts that do not reflect dogs’ natural ecologies nor the real-world situations for which dogs might need to exercise their self-control. That said, these existing tasks provide excellent opportunities for cross-species comparisons of self-control; see [29]. Some researchers have called for a “methodological overhaul” for tasks traditionally used to test self-control in domestic dogs [30,31]. We agree with this and specifically argue that future research should seek to develop new methodologies to assess canine self-control in more naturalistic contexts specific to dogs’ real-world behavioral needs. Exploring canine self-control in ecologically relevant contexts would enable our research to more directly assess and inform dog training practices.

## 5. Conclusions

Our findings highlight what we think is the highly context-specific nature of self-control in dogs, challenging the assumption of many dog trainers that training improves self-control in a domain-general way. While most dogs performed similarly regardless of training level, barn hunt-trained dogs performed worse on the cylinder task compared to untrained pet dogs. We expect this is likely due to interference from task-specific training methods, further supporting our suggestion that self-control training should not be approached as a universal skill, but instead tailored to the specific contexts in which it is most needed. For example, rather than expecting general training behaviors like “wait” to transfer across scenarios, targeted training in real-world contexts—such as refraining from jumping on guests—may better address behavioral goals.

The context specificity of self-control also underscores the need to reevaluate how we typically test it. Current methodologies often involve tasks that are quite disconnected from dogs’ natural behaviors. Developing new, ecologically valid assessments, would not only enhance our understanding of self-control, but also provide more actionable insights for dog training practices. Ultimately this approach could help us bridge the gap between research and practice, ensuring that scientific insights translate into meaningful improvements for dog welfare.

## Figures and Tables

**Table 1:** Summary breed group information.

	Herding	Sporting	Terrier	Toy	Hound	Miscellaneous	Mixed Breeds
Pet	*n* = 9	*n* = 8	*n* = 3	*n* = 5	*n* = 1	*n* = 3	*n* = 20
Agility	*n* = 40	*n* = 13	*n* = 22	*n* = 0	*n* = 0	*n* = 0	*n* = 3
Barn Hunt	*n* = 3	*n* = 3	*n* = 4	*n* = 2	*n* = 2	*n* = 1	*n* = 6
Scent Work	*n* = 4	*n* = 5	*n* = 0	*n* = 1	*n* = 2	*n* = 1	*n* = 1

**Table 2:** Overall cylinder task performance of each training group.

	Cylinder Performance Mean (SD)	Agility Dogs	Barn Hunt Dogs	Scent Work Dogs
Pet Dogs	5.44 (1.36)	*p* = 0.544	*p* = 0.033 *	*p* = 0.409
Agility Dogs	5.06 (1.52)	—	*p* = 0.129	*p* = 0.891
Barn Hunt Dogs	3.91 (2.19)	—	—	*p* = 0.457
Scent Work Dogs	4.79 (1.31)	—	—	—

A star (*) indicates statistical significance.

## Data Availability

The data presented in this study are available on request from the corresponding authors due to privacy reasons.
